# Adolescent Corticosterone and TrkB Pharmaco-Manipulations Sex-Dependently Impact Instrumental Reversal Learning Later in Life

**DOI:** 10.3389/fnbeh.2017.00237

**Published:** 2017-12-07

**Authors:** Elizabeth T. Barfield, Shannon L. Gourley

**Affiliations:** ^1^Department of Pediatrics, Emory University, Atlanta, GA, United States; ^2^Graduate Program in Neuroscience, Emory University, Atlanta, GA, United States; ^3^Yerkes National Primate Research Center, Emory University, Atlanta, GA, United States; ^4^Department of Psychiatry and Behavioral Sciences, Emory University, Atlanta, GA, United States

**Keywords:** juvenile, impulsivity, BDNF, tropomyosin receptor kinase B, 7, 8-dihydroxyflavone

## Abstract

Early-life trauma can increase the risk for, and severity of, several psychiatric illnesses. These include drug use disorders, and some correlations appear to be stronger in women. Understanding the long-term consequences of developmental stressor or stress hormone exposure and possible sex differences is critically important. So-called “reversal learning” tasks are commonly used in rodents to model cognitive deficits in stress- and addiction-related illnesses in humans. Here, we exposed mice to the primary stress hormone corticosterone (CORT) during early adolescence (postnatal days 31–42), then tested behavioral flexibility in adulthood using an instrumental reversal learning task. CORT-exposed female, but not male, mice developed perseverative errors. Despite resilience to subchronic CORT exposure, males developed reversal performance impairments following exposure to physical stressors. Administration of a putative tyrosine kinase receptor B (trkB) agonist, 7,8-dihydroxyflavone (7,8-DHF), during adolescence blocked CORT-induced errors in females and improved performance in males. Conversely, blockade of trkB by ANA-12 impaired performance. These data suggest that trkB-based interventions could have certain protective benefits in the context of early-life stressor exposure. We consider the implications of our findings in an extended “Discussion” section.

## Introduction

Reversal tasks assess the ability of mice or rats to flexibly modify behaviors when reinforcement contingencies change. These tasks are commonly used to model behavioral inflexibility associated with addiction and other disorders in humans (for recent review Izquierdo et al., 2017). Typically, animals are trained to associate specific actions or stimuli with reward (e.g., food), then, the association is modified such that a previously non-predictive contingency could be used to obtain reinforcement, while the original contingency is no longer predictive. Thus, these tasks require animals to inhibit a familiar response strategy and deploy a new strategy to obtain reinforcement.

Behavioral inflexibility in these tasks may result from (Ersche et al., 2008; Jentsch et al., 2002; Schoenbaum et al., 2004) and also *predispose* organisms to (Dalley et al., 2007; Belin et al., 2008; Groman et al., 2009) drug-seeking behaviors. For example, in humans, deficits in reversal learning correlate with the severity of cocaine use (Moreno-López et al., 2015), and a “behavioral disinhibition” trait is associated with later substance use disorders (Tarter et al., 2004; Nigg et al., 2006). Further, rats or mice exhibiting low inhibitory control over impulsive responding more rapidly escalate rates of cocaine self-administration and are more prone to developing drug taking characterized as compulsive (Dalley et al., 2007; Belin et al., 2008; Cervantes et al., 2013). Thus, individual differences in behavioral flexibility and inhibitory control may influence the progression from initial drug use to habitual or compulsive drug-seeking (Cervantes et al., 2013).

Adverse experiences early in life are linked with negative psychiatric outcomes in adulthood, including increased risk for drug use disorders (Fergusson et al., 1996; Kessler et al., 1997; Dube et al., 2003; Green et al., 2010; Afifi et al., 2012) and greater lifetime severity of substance abuse (Hyman et al., 2006; Sacks et al., 2008; Enoch, 2011). Furthermore, several studies report that this association is stronger in women (Widom and White, 1997; MacMillan et al., 2001; Simpson and Miller, 2002; Hyman et al., 2008). The neurobehavioral processes that translate developmental stressor exposure into psychiatric vulnerabilities later in life are incompletely understood, but may involve disruption of prefrontal cortex (PFC)-dependent executive functions, such as behavioral flexibility and inhibitory control (Elton et al., 2014). Indeed, early-life trauma is associated with deficits in inhibitory control (Lewis et al., 2007; Mueller et al., 2010; Skowron et al., 2014; Marshall et al., 2016), and in at least one study, trait impulsivity mediated the relationship between childhood trauma and substance dependence severity in adulthood (Schwandt et al., 2013). Comparing susceptibility to early-life stress-induced inhibitory control deficits between males and females may provide insight into mechanisms of sex differences in risk for stress-related psychopathology.

In the present study, we manipulated levels of the primary glucocorticoid, corticosterone (CORT), and determined long-term consequences in an instrumental reversal task. We exposed mice to exogenous CORT from postnatal day (P) 31–42, corresponding to early adolescence in rodents (Spear, 2000), and a period of considerable structural maturation in the PFC (Shapiro et al., 2017b). Mice were then trained as adults to perform food-reinforced nose poke responses. We found that subchronic CORT exposure induced habit-biased behavior, despite the cessation of CORT exposure, and these findings are reported in Barfield et al. (in press). We then tested mice in an instrumental reversal task, and the results of those tests are reported here.

In some groups, we stimulated tyrosine kinase receptor B (trkB), the high-affinity receptor for brain-derived neurotrophic factor (BDNF), which regulates dendritic spine structure and function (Yoshii and Constantine-Paton, 2010). BDNF-trkB systems are impacted by stress (Gray et al., 2013; Numakawa et al., 2013), implicated in addiction-related behaviors (Li and Wolf, 2015), and significantly modify reward-related decision making (Pitts et al., 2016). Loss of neurotrophic support following prolonged exposure to elevated glucocorticoids is thought to contribute to structural and functional alterations in the PFC that are associated with stress-related psychopathology (Duman et al., 1997). Moreover, recent findings implicate down-regulation of BDNF-trkB signaling in the long-term behavioral consequences of adolescent stress exposure (Xu et al., 2016; Zhang et al., 2017; Barfield et al., in press). Thus, we hypothesized that stimulating trkB may have protective benefits in animals exposed to CORT during adolescence, blocking enduring behavioral deficits.

We report sex-dependent long-term consequences of both CORT and trkB manipulations in an instrumental reversal task. Namely, a history of early-adolescent CORT exposure in females, but not males, induced perseverative responding. Despite apparent resilience to early-adolescent CORT, males developed behavioral inflexibilities following repeated stressor exposure or trkB blockade during early adolescence. Further, a putative trkB agonist improved performance in both sexes. We consider the implications of our findings in an extended “Discussion” section.

## Materials and Methods

### Subjects

Subjects were male and female wild-type C57BL/6 mice (Jackson Labs, Bar Harbor, ME, USA) or mice expressing *thy1*-derived yellow fluorescent protein (YFP; Feng et al., 2000) that were fully back-crossed onto a C57BL/6 background. Mice were not handled, other than for routine veterinary care, until P31. Mice were group-housed, maintained on a 12-h light cycle (0700 on) and provided food and water *ad libitum* except during instrumental conditioning when body weights were maintained at 90%–93% of baseline to motivate responding. Animal numbers for each experiment are indicated in the respective figure captions. This study was carried out in accordance with the recommendations of the Guide for the Care and Use of Laboratory Animals. The protocol was approved by the Emory University IACUC.

### CORT Exposure

CORT hemisuccinate (4-pregnen-11β 21-DIOL-3 20-DIONE 21-hemisuccinate; Steraloids) was dissolved in tap water (25 μg/mL free base; Gourley et al., 2008a,b, 2012b). CORT-exposed mice were given CORT in place of normal drinking water, while control mice consumed tap water. CORT solutions were changed every 3 days. Water bottles were weighed daily, and mice weighed every other day to calculate average doses (~5–9 mg/kg/day) of CORT. Mice were exposed to CORT or water from P31 to 42, corresponding to early adolescence in rodents (Spear, 2000). After a 2-week washout period, when mice reached young adulthood (P56), instrumental conditioning began. Timelines are in the figures.

### Forced Swim Stress

Mice were exposed to forced swim stress daily from P31 to 42. Mice were placed in a glass cylinder (24 cm × 15.5 cm diameter) filled with 25°C water in a dimly lit room. After 6 min, mice were dried in a warm cage lined with paper towels, then returned to the home cage. Water was changed between mice. Control mice were handled but not exposed to swim stress.

### Instrumental Conditioning

**Figure 1:**
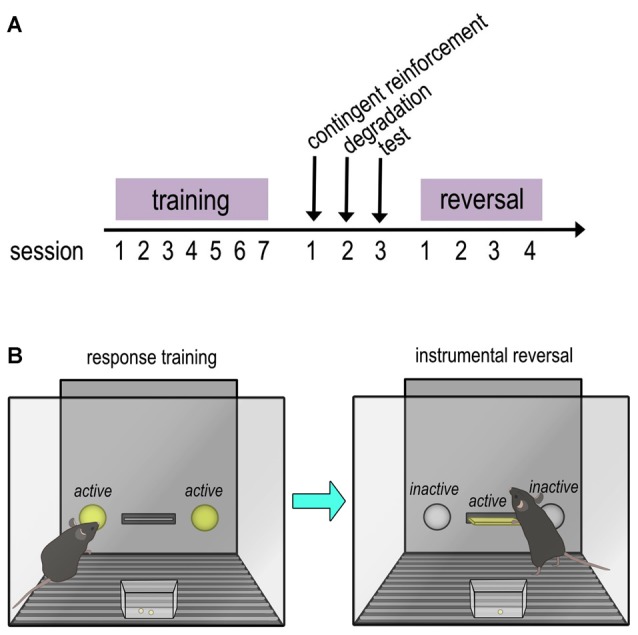
Timeline and schematic of instrumental training and reversal task. **(A)** Timeline of training and tasks. Mice were trained to nose poke in operant conditioning chambers for food reinforcers. Next, an action-outcome contingency degradation task was performed, in which the contingency between an action and outcome are “degraded” by providing food pellets independently of the mouse’s actions. The data from these tests are reported in Barfield et al. (in press). An instrumental reversal task followed, and the data from these tests are reported in the present manuscript. **(B)** Schematic of response training and instrumental reversal. In the instrumental reversal task, the reinforced response was “reversed” from a nose poke on the sides of the chamber to a lever press located in the center of the chamber that had not previously been available. Acquisition of the newly reinforced response (lever press) and inhibition of the previously reinforced response (nose poke) were measured over multiple sessions.

Mice were trained to nose poke for food reinforcement (20 mg grain-based pellets; Bio-Serv, Flemington, NJ, USA) using Med Associates conditioning chambers equipped with two nose-poke recesses, a retractable lever, and a food magazine. Responding on each of two nose-poke recesses was reinforced using a fixed ratio 1 (FR1) schedule of reinforcement; 30 reinforcers were available for responding on each aperture, resulting in 60 reinforcers/session. Sessions ended when mice acquired all 60 reinforcers or at 70 min. Five to seven daily training sessions were conducted. Response acquisition curves represent both nose poke responses/min. Mice were then tested in an instrumental contingency degradation task, followed by a reversal task (see timeline in Figure 1A). Given our focus on reversal conditioning here, only reversal performance is shown.

#### Instrumental Reversal Test

In this task, the reinforced response was “reversed” to a lever press; there were no consequences for generating the previously reinforced nose poke response (Figure 1B). Lever pressing was reinforced using an FR1 schedule, with one 25-min session/day for four consecutive days. Lever press rates reflect acquisition of a new response, while nose poke rates reflect inhibition of the previously reinforced response. The percent of correct responses made during session 1 was calculated by dividing total lever presses by total responses (lever presses + nose pokes).

### Drugs (Dosing and Timing)

Male and female mice were administered (*i.p*.) the putative trkB agonist, 7,8-dihydroxyflavone (7,8-DHF; Sigma; 3 mg/kg; dissolved in 17% DMSO and saline; Zhang et al., 2014), or vehicle daily from P39 to 47, overlapping with the end of the adolescent CORT exposure period. This period is marked by significant pruning of dendritic spines on excitatory pyramidal neurons in the mouse orbital PFC (oPFC; Shapiro et al., 2017b). Additionally, expression levels of trkB in the oPFC increase during this time (Shapiro et al., 2017b), potentially facilitating activity-dependent refinement of synaptic connections and stabilizing synapses that are not pruned. This period was also determined based on prior work (Barfield et al., in press).

The trkB antagonist, ANA-12 (Sigma; 0.5 mg/kg, 1% DMSO), or vehicle was administered (*i.p*.) daily from P31 to 42, to match the period of adolescent CORT exposure.

### Statistical Analyses

Two-tailed statistical analyses with α ≤ 0.05 were performed using SPSS. Response rates were compared by two-factor (group × session) or three-factor (CORT × 7,8-DHF × session) mixed analysis of variance (ANOVA) with session as a within-subjects (repeated measure) factor. Tukey’s *post hoc* tests were used following interactions or main effects between greater than two groups, and results are indicated graphically. The percent of responses that were reinforced (“correct”) were compared by one-factor or two-factor (CORT × 7,8-DHF) ANOVA or Student’s *t*-tests. In additional comparisons, CORT-exposed mice were split into “vulnerable” and “non-vulnerable” groups based on a median split of percent correct values from session 1 of the instrumental reversal task. Throughout, values >2 SDs from the mean were excluded.

## Results

### Early-Adolescent Corticosteroid Exposure in Females Impairs Response Inhibition in Adulthood

**Figure 2:**
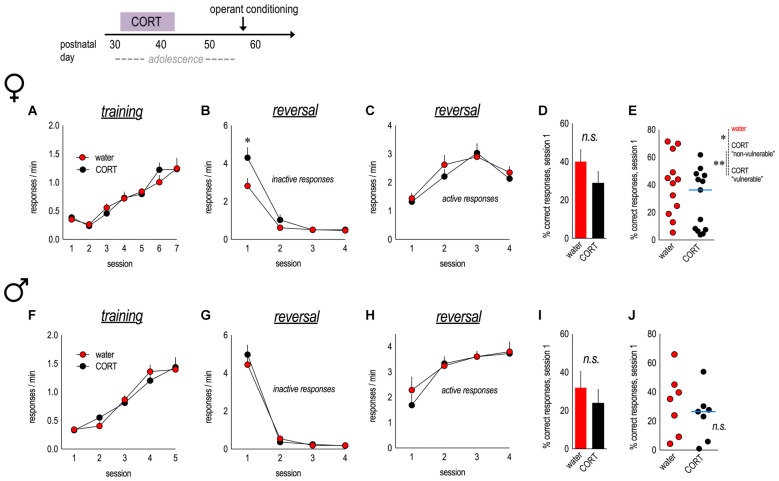
Corticosterone (CORT) exposure in adolescent females induces reversal errors in adulthood. An experimental timeline is at top. **(A)** Female mice with a history of CORT exposure were trained to nose poke for food reinforcers, with no group differences. **(B)** In an instrumental reversal task, CORT-exposed mice made more perseverative errors, generating the previously-reinforced response. **(C)** CORT did not impact the acquisition of the new lever press response. **(D)** The percent of correct responses made during session 1 did not differ between groups. **(E)** However, a median split revealed that CORT-exposed females separated into two distinct subgroups; the “vulnerable” group made significantly fewer correct responses. *n* = 12–13/group. **(F)** Male mice exposed to CORT during adolescence acquired the nose poke responses without group differences. **(G)** A history of early-adolescent CORT exposure did not impact inhibition of the previously reinforced, now-inactive, response. **(H)** Acquisition of the newly reinforced response was also not impacted. **(I,J)** The percent of correct responses made during session 1 also did not differ between groups. *n* = 7/group. Symbols and bars represent means + SEMs, except for scatter plots, where symbols represent individual mice, and blue lines indicate the median. *p ≤ 0.05. **p ≤ 0.001.

**Figure 3:**
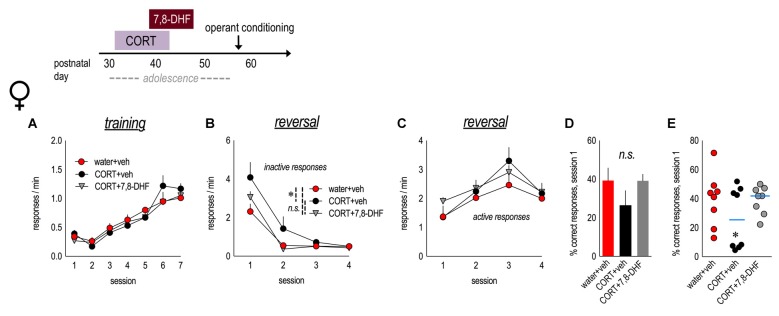
Tyrosine kinase receptor B (TrkB) stimulation blocks perseverative errors in CORT-exposed female mice. Experimental timeline is at top. Female mice were exposed to CORT from P31 to 42 and administered the trkB agonist, 7,8-dihydroxyflavone (7,8-DHF), from P39 to 47. **(A)** As adults, mice were trained to nose poke for food reinforcers, with no differences between groups. **(B)** CORT-exposed mice made more perseverative errors, and 7,8-DHF mitigated this effect. **(C)** Groups generated the newly reinforced response at similar rates. **(D)** There were no gross effects on the percent of correct responses made during session 1. **(E)** CORT-exposed mice were divided into two subgroups on the basis of a median split of percent values, revealing two populations, including a subgroup that made significantly fewer correct responses. 7,8-DHF eliminated these differences. *n* = 8/group. Symbols and bars represent means + SEMs. *p ≤ 0.05.

**Figure 4:**
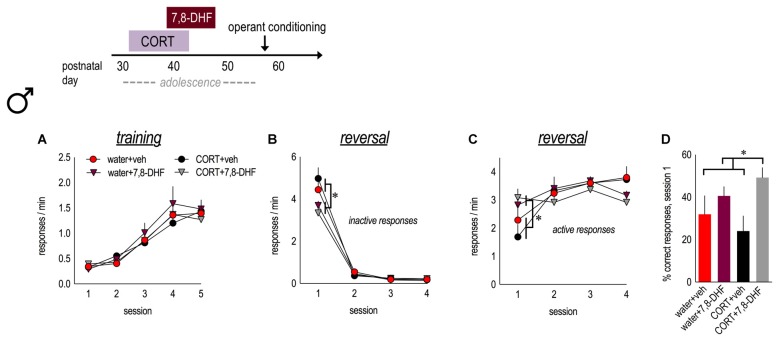
TrkB stimulation during adolescence enhances behavioral flexibility in adulthood. Experimental timeline is at top. Male mice were exposed to CORT from P31 to 42 and administered the trkB agonist, 7,8-DHF, from P39 to 47. **(A)** Mice acquired the nose poke responses in adulthood, with no group differences. **(B)** In both water and CORT-exposed mice, 7,8-DHF reduced perseverative errors in the first reversal session. **(C)** Further, 7,8-DHF increased active response rates on the first day of reversal. **(D)** 7,8-DHF also increased the percent of correct responses made during session 1 (asterisk signifies a main effect of 7,8-DHF). *n* = 7/group. Symbols and bars represent means + SEMs. *p ≤ 0.05.

We exposed female and male mice to CORT in the drinking water from P31 to 42, equivalent to early adolescence in humans (Spear, 2000). Some mice also received daily *i.p*. injections of vehicle (17% DMSO and saline) or 7,8-DHF (3 mg/kg) from P39 to P47. In the interest of clarity, only vehicle-treated and non-injected mice are shown in Figure 2, and then all injected groups are represented in Figures 3, 4, respectively.

As adults, female mice acquired the nose poke responses without group differences (main effect and interaction *F*s < 1; Figure 2A). When the response requirement was “reversed” to a lever press, females with a history of adolescent CORT exposure made more perseverative errors than control mice—responding on the previously reinforced nose poke apertures despite non-reinforcement—during session 1 (interaction *F*_(3,69)_ = 4.0, *p* = 0.01; Figure 2B). Meanwhile, acquisition of the newly reinforced lever response was unaffected (main effect and interaction *F*s < 1; Figure 2C).

We also calculated the percentage of total responses that were reinforced during session 1. While this measure did not significantly differ between groups (*t*_(23)_ = 1.3, *p* = 0.2; Figure 2D), mice with a history of CORT exposure qualitatively appeared to segregate into two groups. As an exploratory analysis, we applied a median split to CORT mice. CORT-exposed “vulnerable” mice made significantly fewer correct responses relative to both control and CORT-exposed “non-vulnerable” mice (main effect group *F*_(2,22)_ = 11.1, *p* < 0.001; Figure 2E). Thus, in females, a history of CORT exposure during early adolescence increases perseverative errors, and considerable individual differences are noted.

We repeated this experiment in male mice. Mice acquired the food-reinforced nose poke responses in adulthood, without group differences (main effect and interaction *F*s < 1; Figure 2F). In contrast to females, early-adolescent CORT exposure in males did not impact response reversal (errors, main effect and interaction *F*s < 1; Figure 2G; lever response acquisition, main effect and interaction *F*s < 1; Figure 2H). There were no differences in the percentage of responses that were reinforced (*t*_(12)_ = 0.8, *p* = 0.5; Figure 2I), and a median split of values revealed no differences between control mice and either of the two CORT-exposed subgroups (main effect group *F*_(2,11)_ = 2.0, *p* = 0.2; Figure 2J). Thus, females may be especially vulnerable to the long-term effects of CORT exposure during early adolescence on response inhibition.

### TrkB Stimulation Blocks CORT-Induced Errors

We attempted to block the long-term behavioral consequences of adolescent CORT exposure in females by administering 7,8-DHF (3 mg/kg) during mid-adolescence (P39–47). A 7,8-DHF-only group was omitted to conserve animal usage. We found no group differences in response acquisition (main effect and interaction *F*s < 1; Figure 3A). As before, mice with a history of adolescent CORT exposure generated more non-reinforced responses in reversal (main effect group *F*_(2,21)_ = 3.6, *p* = 0.046; session × group interaction *F*_(6,63)_ = 2.1, *p* = 0.07), and treatment with 7,8-DHF mitigated this impairment in response inhibition (Figure 3B). Acquisition of the newly reinforced lever press did not differ (main effect and interaction *Fs ≤ 1;* Figure 3C). Groups did not differ in the percentage of responses that were reinforced during session 1 (main effect group *F*_(2,21)_ = 1.5, *p* = 0.3; Figure 3D). Further, treatment with 7,8-DHF eliminated the existence of “vulnerable” and “non-vulnerable” populations within the CORT-exposed group (ANOVA with CORT+vehicle and CORT+7,8-DHF mice separated into two subgroups based on a median split (five groups total): main effect group *F*_(4,19)_ = 7.9, *p* = 0.001; Figure 3E). Thus, a trkB agonist occludes long-term deficits in response inhibition following early-life CORT exposure.

### TrkB Stimulation in Adolescence Improves Behavioral Flexibility in Adulthood

Male mice exposed to water or CORT during early adolescence were also administered either vehicle or 7,8-DHF. As adults, all mice acquired the nose poke responses for food reinforcement, without group differences (main effect and interaction *F*s ≤ 1.2; Figure 4A). In instrumental reversal, a history of CORT exposure did not impact response inhibition (*F*s < 1) or acquisition of the newly reinforced response (*F*s < 1), as described above. Nevertheless, 7,8-DHF reduced perseverative responding during session 1 in both control and CORT-exposed animals (session × 7,8-DHF interaction *F*_(3,72)_ = 7.1, *p* < 0.001; Figure 4B) and increased responding on the newly reinforced lever in session 1 (session × 7,8-DHF interaction *F*_(3,72)_ = 6.6, *p* = 0.001; Figure 4C). 7,8-DHF also increased the percentage of responses that were correct during session 1 (main effect *F*_(1,24)_ = 7.7, *p* = 0.01; CORT × 7,8-DHF interaction* F*_(1,24)_ = 1.8, *p* = 0.2; Figure 4D), further indicating that stimulation of trkB during adolescence enhances behavioral flexibility in adulthood, well beyond the period of drug treatment.

### Adolescent Stressor Exposure and TrkB Inhibition Impair Reversal Performance

**Figure 5:**
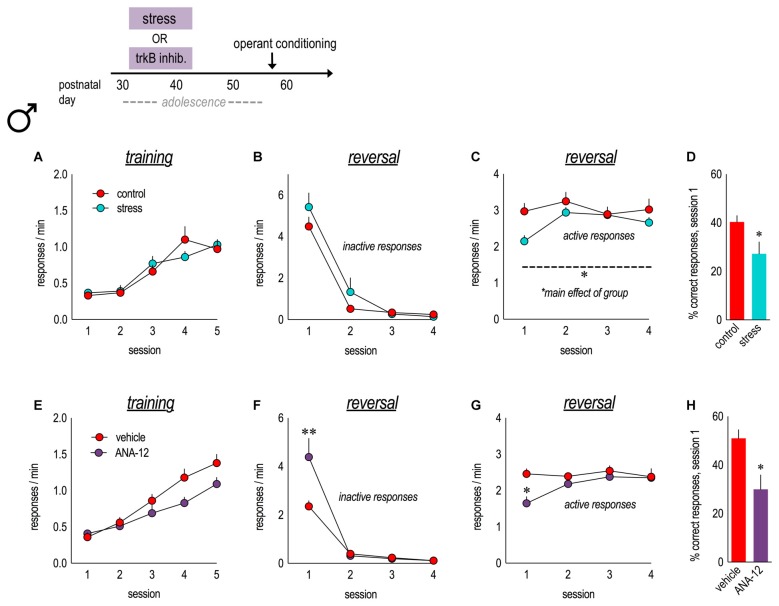
Physical stressors and pharmacological inhibition of trkB in adolescence impair instrumental reversal in adulthood. Experimental timeline is at top. **(A)** A history of stressor exposure did not impact responding for food reinforcement during training in adulthood. **(B)** In an instrumental reversal task, both groups performed the now-inactive nose poke response at similar rates. **(C)** By contrast, stressor-exposed mice performed the now-active lever press response at lower rates. **(D)** Adolescent stressor exposure also reduced the percent of correct responses made during session 1. *n* = 9–10/group. **(E)** In parallel experiments, mice received daily injections of the trkB antagonist, ANA-12, from P31 to 42. Mice learned to nose poke for food reinforcement in adulthood, with no group differences. **(F)** TrkB blockade during adolescence increased perseverative errors during the first reversal session. **(G)** ANA-12 also reduced responding on the now-active lever during session 1. **(H)** ANA-12-treated mice made fewer correct responses during session 1. *n* = 7/group. Symbols and bars represent means + SEMs. *p ≤ 0.05. **p ≤ 0.001.

The apparent resilience of male mice to early-adolescent CORT exposure was somewhat surprising, so we exposed another group of males to swim stress during early adolescence (daily from P31 to 42). In adulthood, we detected no group differences in the acquisition of the nose poke responses (main effect *F* < 1; interaction *F*_(4,68)_ = 1.8, *p* = 0.1; Figure 5A). When mice were required to shift responding to a previously non-reinforced lever, control and stressor-exposed mice did not differ in responding on the previously reinforced nose poke apertures (main effect *F*_(1,16)_ = 1.3, *p* = 0.3; interaction *F*_(3,48)_ = 1.3, *p* = 0.3; Figure 5B). However, mice with a history of adolescent stressor exposure responded less on the newly reinforced lever (main effect *F*_(1,16)_ = 8.1, *p* = 0.01; session × group interaction *F*_(3,48)_ = 1.2, *p* = 0.3; Figure 5C), making fewer reinforced responses during session 1 (*t*_(17)_ = 2.3, *p* = 0.04; Figure 5D). Thus, stressor exposure in early adolescence impairs the ability of male mice to develop novel response strategies in adulthood. This deficiency is similar to those following lateral oPFC (loPFC) ablation (Gourley et al., 2010; summarized in Table 1).

**Table 1:** Failures in response acquisition and errors of perseveration in an instrumental reversal task can be dissociated.

Manipulation	Region	Sex	Reference
**Impairments of response acquisition**			
Early-adolescent forced swim stress	n/a	M	Figure 5C
Early-adolescent trkB antagonism	n/a	M	Figure 5G
Lesion	loPFC	M	Gourley et al. (2010)
Inhibition of Abl-family kinases	loPFC	M	Gourley et al. (2012a)
*Bdnf* knockdown	loPFC	M	Gourley et al. (2013a)
Disconnection of the loPFC and striatum	loPFC	M	Gourley et al. (2013a)
**Impairments of response inhibition**			
Early-adolescent CORT exposure	n/a	F	Figure 2B
Early-adolescent trkB antagonism	n/a	M	Figure 5F
Lesion	moPFC+loPFC	M	Boulougouris et al. (2007)
Lesion	moPFC	M	Gourley et al. (2010)

*Errors of perseveration (repeatedly performing an “old” response, despite reversal) or response acquisition (failing to develop a new strategy) can be dissociated in reversal tasks that rely on spatial or instrumental learning and memory. Here we detected failures in response acquisition and errors of perseveration (highlighted in blue). These deficits are associated with manipulations of the lateral oPFC (loPFC) and medial oPFC (moPFC), respectively (highlighted in gray)*.

Prolonged stressor or glucocorticoid exposure can down-regulate *Bdnf* or BDNF-trkB signaling in the PFC, and these alterations are associated with impairments in PFC-dependent behavioral tasks (Gourley et al., 2009a, 2012b; Xu et al., 2016; Zhang et al., 2017). Thus, we also tested whether inhibiting trkB during early adolescence would induce reversal deficits in male mice.

Male mice received the trkB antagonist, ANA-12 (0.5 mg/kg), daily from P31 to 42, matching the period of early-adolescent CORT exposure. As adults, mice acquired the nose poke responses for food reinforcement, without group differences (main effect *F*_(1,12)_ = 3.6, *p* = 0.08; interaction* F*_(4,48)_ = 2.2, *p* = 0.09; Figure 5E). ANA-12 impaired response inhibition (interaction *F*_(3,33)_ = 10.4, *p* < 0.001; Figure 5F) and also acquisition (interaction *F*_(3,30)_ = 4.1, *p* = 0.01; Figure 5G) during session 1. ANA-12-treated mice made fewer correct responses overall during session 1 (*t*_(12)_ = 3.1, *p* = 0.01; Figure 5H). Thus, trkB inhibition during adolescence recapitulated the effects of CORT exposure in females (perseverative responding) and also stressor exposure in males (response acquisition impairments). Our findings are summarized in Table 1.

## Discussion

Adolescents who experience chronic stress have higher incidences of stress-related psychiatric illnesses and behaviors associated with addiction as adults. These behaviors may result in part from disruption of the PFC-dependent ability to flexibly modify behavior when environmental contingencies change (Elton et al., 2014; Zhang et al., 2016; see for review Watt et al., 2017). BDNF signaling through trkB regulates cellular maturational processes occurring in the PFC during adolescence (Xu et al., 2000; Shapiro et al., 2017b), and is disrupted by exposure to stress or elevated glucocorticoids (Bath et al., 2013; Numakawa et al., 2013; Suri and Vaidya, 2013). Using an oPFC-dependent instrumental reversal task in mice (see Gourley et al., 2010), we evaluated the long-term consequences of adolescent exposure to CORT, physical stress, trkB stimulation, or trkB inhibition, on behavioral flexibility. In females, adolescent CORT exposure increased perseverative errors in adulthood, and this was blocked by a trkB agonist. By contrast, behavioral flexibility was not impacted by CORT exposure in males, but was enhanced by trkB stimulation, as in females. Males did, however, develop reversal deficits in adulthood following repeated stressor exposure or trkB antagonism during adolescence. These findings add to evidence that adverse events or elevated glucocorticoid levels during adolescence can impair behavioral flexibility later in life. Moreover, our data suggest that trkB-based interventions may prevent adolescent stress-related behavioral impairments later in life.

### Long-Term, Sex-Dependent Behavioral Consequences of Adolescent CORT Exposure

The majority of rodent studies examining the long-term neurobehavioral effects of early-life adversity have utilized male subjects, despite evidence for sex differences in the prevalence and severity of stress-related psychiatric illnesses (Kessler et al., 1993; MacMillan et al., 2001; Holbrook et al., 2002; Kuehner, 2003; Becker et al., 2012; Fattore et al., 2014). We report that CORT dissolved in the drinking water of mice during a period equivalent to early adolescence in humans (P31–42; Spear, 2000) impaired performance in an instrumental reversal task in adulthood in female mice, but not males. Specifically, adolescent CORT impaired the inhibition of a previously reinforced response, increasing perseverative errors. Similarly, exposure to stress-level cortisol in young-adult or older-adult female squirrel monkeys increases response inhibition errors in a detour-reaching task that assesses the ability to inhibit prepotent responses when reinforcement contingencies change (Lyons et al., 2000).

We also calculated the percent of responses that were reinforced (“correct”) during the first reversal session, revealing a “CORT vulnerable” subgroup (approximately half of the group) with significantly impaired behavioral flexibility relative to a CORT-exposed “non-vulnerable” group and control mice. What mediates this vulnerability is unclear, but one factor may be dopamine D2-family receptors (D2, D3, D4). D2/3 antagonists disrupt reversal performance in monkeys and rodents (Ridley et al., 1981; Kruzich and Grandy, 2004; Floresco et al., 2006; Lee et al., 2007; De Steno and Schmauss, 2009), and variation in D2 receptor density in the ventral PFC (including oPFC; Gourley et al., 2009b) and midbrain (Laughlin et al., 2011) correlates with behavioral flexibility. Furthermore, adolescent stress exposure decreases PFC D2 expression in adulthood (Wright et al., 2008). Whether vulnerabilities to corticosteroid-related modifications in dopamine D2 levels influence the behavioral patterns reported here could be tested in future experiments.

In contrast to females, males exposed to early-adolescent CORT failed to develop instrumental reversal impairments in adulthood. However, some studies report long-term deficits in reversal learning and extradimensional set-shifting (EDS; a medial PFC (mPFC)-dependent function) in male rodents following social stress during early adolescence (Snyder et al., 2015; Zhang et al., 2016). Accordingly, daily forced swim stress during early adolescence (P31–42) impaired the acquisition of the “reversed” response contingency here, resembling the effects of loPFC lesions (Gourley et al., 2010). These findings suggest that in males exposed to repeated stress during adolescence, components of the stress response besides elevations in CORT (e.g., increased noradrenergic tone) may be responsible for response acquisition deficiencies. Indeed, under conditions of chronic stress in adult rats, stress-induced release of norepinephrine in the mPFC impairs certain forms of behavioral flexibility, while noradrenergic receptor blockade in the mPFC prevents stress-induced deficits (Jett and Morilak, 2013). On the other hand, 3 weeks of CORT exposure in adolescent male mice causes errors in a similar instrumental reversal task (Shapiro et al., 2017a), indicating that males are not entirely resilient to CORT.

One possibility is that higher concentrations of CORT would have triggered reversal deficiencies in males. Females have higher basal CORT levels than males, an effect that develops during puberty (Netherton et al., 2004; McCormick and Mathews, 2007; Stroud et al., 2011). Because puberty in rodents typically occurs around P35 in females and P40 in males (Korenbrot et al., 1977; Evans, 1986), administering exogenous CORT during a time when endogenous levels in females are increasing may result in higher total CORT levels in females. While we did not measure blood serum CORT in these experiments, it is possible that recapitulating “female-like” CORT levels in males may have induced comparable behavioral inflexibility.

Because the PFC undergoes significant structural remodeling during adolescence, and PFC-dependent cognitive functions continue to develop throughout this period, prolonged stressor or CORT exposure during adolescence may be more impactful than exposure in adulthood. Consistent with this possibility, male rats exposed to 5 days of social defeat stress during early adolescence (P28–32), then housed in isolation, exhibit deficits in EDS and reversal learning in an attentional set-shifting task 6 weeks later. However, rats exposed to the same stress in mid-adolescence (P38–47) or adulthood (P70–79) do not show these cognitive impairments (Zhang et al., 2016). Furthermore, adolescent stress- or CORT-induced alterations in PFC neuronal morphology, like behavioral deficits, can persist into adulthood. For example, social isolation stress during early adolescence (P30–35) in male rats reduces synaptic density in the mPFC in adulthood (Leussis et al., 2008), suggestive of a long-term loss of dendritic spines, which house the majority of excitatory synapses in the brain. Gourley et al. (2013b) also reported dendritic spine loss on pyramidal neurons in the oPFC immediately following CORT exposure during adolescence (P35–56) that is still evident 1 week after the cessation of CORT. Meanwhile, CORT-induced spine changes in the infralimbic mPFC, hippocampus, and amygdala recover. Consistent with these findings in rodents, early-life adversity in humans is associated with reduced oPFC volume and morphological alterations in adulthood (Holz et al., 2015; Teicher and Samson, 2016). Our findings add to these and other studies reporting long-term consequences of stressor or CORT exposure during early adolescence.

### TrkB Manipulations in Adolescence Influence Behavioral Flexibility in Adulthood

One mechanism by which CORT or stressor exposure during adolescence may impact PFC-dependent behaviors later in life is by reducing BDNF-dependent stimulation of trkB (Duman et al., 1997; Blugeot et al., 2011). Consistent with this possibility, the trkB agonist 7,8-DHF blocked perseverative responding in females with a history of adolescent CORT exposure. Additionally, while a subgroup of vehicle-treated CORT-exposed females was especially vulnerable to impairments in behavioral flexibility, 7,8-DHF eliminated these individual differences, and in males, 7,8-DHF improved performance above control levels.

How might 7,8-DHF have long-term behavioral consequences? One possibility is that it prevents CORT-induced disruptions in PFC synaptic maturation by augmenting or restoring trkB activity. BDNF and trkB increase in adolescence/young adulthood (Webster et al., 2002; Shapiro et al., 2017b), and BDNF-trkB signaling is critical for the maintenance of stabile dendritic spines in the postnatal brain (Vigers et al., 2012). Chronic stress or CORT exposure can reduce PFC BDNF and trkB (e.g., see Gourley et al., 2009a, 2012b), potentially altering the trajectory of dendritic spine maturation (Leussis and Andersen, 2008; Eiland et al., 2012) and behavioral outcomes. Consistent with this possibility, we found that administration of the trkB antagonist, ANA-12, during early adolescence impaired behavioral flexibility in adulthood.

Our trkB antagonism experiments were performed in males, but it is worth noting that prior reports indicate that knockdown of *Bdnf* beginning in the preadolescent period or in adulthood in female mice produces behavioral consequences that are comparable to, or more severe than, those in male mice. For example, Vigers et al. (2012) report that forebrain-specific knockdown of *Bdnf* (progressive knockdown begins at ~P21) induces depressive-like immobility in the forced swim test and contextual fear generalization in both male and female adult mice. However, Monteggia et al. (2007) report that this knockdown strategy results in depression-like behavior in female, but not male, mice. Similarly, inducible knockdown of *Bdnf* in adulthood increases susceptibility to stress-induced depression-like behavior in female mice only (Autry et al., 2009).

Recent findings in addition to ours interestingly implicate BDNF-trkB in the long-term behavioral consequences of early-adolescent stress. Male rats exposed to chronic mild stress (CMS) during early adolescence (P28–37) exhibit impairments in EDS and decreased levels of BDNF protein, the ratio of p-ERK42/44/ERK42/44, and p-CREB in the mPFC 6 weeks after stressor exposure (Zhang et al., 2017). Additionally, EDS is correlated with p-ERK/ERK ratios in mPFC, and the antidepressant duloxetine ameliorates stress-induced alterations in EDS and mPFC BDNF levels in tandem.* Impairing* BDNF-trkB signaling can also alter PFC-dependent functions and drug-taking behaviors. For example, mutant mice with disruption of promoter IV-driven (activity-dependent) BDNF expression are impaired in a spatial reversal task (Sakata et al., 2013). Knock-in mice expressing a homolog of the human *BDNF* gene polymorphism *Val66Met*, which decreases the activity-dependent release of BDNF (Chen et al., 2004), develop excessive and compulsive alcohol drinking despite adverse consequences, and this phenotype is reversible by a trkB agonist (Warnault et al., 2016). Furthermore, early-life stress decreases *Bdnf* exon IV mRNA in the PFC and increases cocaine-induced conditioned place preference (CPP) in periadolescent (P45) mice (Viola et al., 2016). Interestingly, higher CPP scores correlate with *Bdnf* expression, with higher place preference associated with lower *Bdnf*. Thus, by disrupting trkB signaling, early-life stress may increase vulnerability to compulsive drug taking.

### Broader Considerations: Sex Differences in Reversal Performance

Here, we found that adolescent CORT-exposed females and adolescent stress-exposed males exhibited impairments in reversal performance in adulthood; however, the specific behavioral impairments were sexually dimorphic—females failed to inhibit the previously reinforced response, while males were unable to acquire a newly reinforced response (summarized in Table 1). In the context of addiction, poor control over perseverative behavior could modulate the progression from recreational drug use to abuse and dependence, increase relapse rates, and impair treatment response (Carroll et al., 2011). Meanwhile, deficits in the acquisition of a “reversed” response contingency could be comparable to impairments in the ability to adjust behavior according to positive feedback and potentially, to substitute new healthy behaviors for maladaptive ones.

The association between early-life trauma and the risk for, and severity of, drug dependence is stronger in women (Widom and White, 1997; MacMillan et al., 2001; Simpson and Miller, 2002; Hyman et al., 2008), and at least one study reports that a greater proportion of stimulant-dependent women, compared to men, have clinically significant “disinhibition” scores prior to stimulant abuse (Winhusen and Lewis, 2013). In that same study, physical abuse was associated with greater disinhibition in women, but not men. Thus, females may be more vulnerable to chronic stress-induced inhibitory control deficits, in line with our findings. Further, Elton et al. (2014) report that childhood maltreatment results in enduring, sex-dependent changes in the functional connectivity of a network mediating inhibitory behavioral control, also in line with sex-dependent effects of adolescent CORT exposure on perseverative responding here. Future work should examine whether adolescent CORT-induced deficits in response inhibition increase vulnerability to compulsive drug taking later in life. CORT exposure during adolescence increases cue-induced reinstatement of ethanol seeking in adult female, but not male, rats (Bertholomey et al., 2016), suggesting that females are especially vulnerable to adolescent CORT-induced neurobehavioral changes that increase susceptibility to drug relapse later in life.

Our findings may also point to sexually dimorphic stress-related vulnerabilities in specific brain regions, given that lesions of the medial oPFC (moPFC) impair response inhibition in this task (like CORT-exposed females), while loPFC lesions impair response acquisition (like stressor-exposed males; Gourley et al., 2010). These patterns are interesting when considered alongside studies reporting structural changes in the oPFC of substance-dependent males and females. For example, cocaine-dependent females and males exhibit decreased cerebral blood flow in the moPFC and loPFC, respectively (Adinoff et al., 2006). Adolescent females with substance dependence exhibit decreased cortical thickness and gray matter volume in regions involved in inhibitory control, decision-making, reward, and risk-taking, including the moPFC and dorsolateral PFC (DLPFC; Dalwani et al., 2015; Boulos et al., 2016), while substance-dependent adolescent males show decreased gray matter volume in DLPFC (Dalwani et al., 2011). Regner et al. (2015) report decreased oPFC gray matter volume in abstinent stimulant-dependent women, but not men. However, in a population of predominantly male treatment-seeking individuals with alcohol dependence, loPFC surface area and volume were smaller in individuals who subsequently relapsed in a 1-year period compared to individuals who remained abstinent (Cardenas et al., 2011; Durazzo et al., 2011). Together, these findings suggest that PFC subregions disrupted by both adolescent stress or glucocorticoid exposure and drugs of abuse may be sex-dependent (potentially: loPFC in males and both moPFC and loPFC in females; see Table 1).

## Conclusion

PFC dysfunction and associated deficits in inhibitory control and behavioral flexibility are hallmarks of stress-related illnesses, including addiction. Substance-dependent individuals report loss of control over drug use and have difficulty modifying behaviors when reward contingencies change, potentially contributing to persistent drug seeking despite adverse consequences (Garavan and Hester, 2007; Everitt and Robbins, 2016). Being able to implement new strategies to promote behavioral change and control impulsive responses may be critical to treatment response—among cocaine-dependent individuals undergoing cognitive-behavioral therapy, higher impulsivity/risk-taking before treatment is associated with poorer treatment retention and higher relapse rates (Carroll et al., 2011).

Substance-dependent individuals exhibit structural and functional alterations in the oPFC, including blood flow abnormalities (London et al., 2000; Adinoff et al., 2006), decreased gray matter volume (Matochik et al., 2003; Tanabe et al., 2009; Dalwani et al., 2015), and decreased cortical thickness (Boulos et al., 2016). In animal models, adolescent CORT and cocaine exposure result in long-term changes in oPFC neuron structure (Gourley et al., 2012a, 2013b; DePoy et al., 2014, 2016, 2017) that are associated with maladaptive behaviors symptomatic of addiction (Lucantonio et al., 2012). Further, poor inhibitory control may be a *predisposing* *factor* for addiction (Jentsch and Taylor, 1999; Tarter et al., 2004; Groman et al., 2009; Moffitt et al., 2011), as is a history of early-life adversity (Fergusson et al., 1996; Kessler et al., 1997; Dube et al., 2003; Green et al., 2010; Afifi et al., 2012). These and other findings have led to the hypothesis that early-life adversity may affect addiction liability by impairing oPFC-mediated inhibitory control. That is, in individuals with a history of stressor exposure during sensitive developmental periods like adolescence, repeated drug use may further impair inhibitory control, exacerbating the development, persistence, and severity of addiction. Indeed, social stress during adolescence in rats causes binge-like cocaine self-administration in adulthood (Burke and Miczek, 2015).

Using an oPFC-dependent instrumental reversal task, we find that exposure to CORT during early adolescence induces perseverative errors in female, but not male, mice in adulthood. Further, intervention with a trkB agonist in mid-adolescence, when dendritic spines in the PFC undergo activity-dependent pruning or stabilization (Petanjek et al., 2011; Gourley et al., 2012a; Selemon, 2013; Chung et al., 2017) corrected decision-making strategies in CORT-exposed mice. With the caveat that the sexes were tested in independent cohorts here—precluding direct comparisons between them—we nevertheless argue that further related research may advance our understanding of how sex differences in the neurobehavioral response to adolescent adversities contribute to sex differences in vulnerability to, and clinical course of, stress-related illnesses. Our findings additionally suggest that pharmacotherapies augmenting trkB may ameliorate the enduring consequences of stressful life events occurring in adolescence.

## Author Contributions

ETB performed the experiments, analyzed the data and wrote the manuscript. ETB and SLG designed the experiments, interpreted the data and edited the manuscript.

## Conflict of Interest Statement

The authors declare that the research was conducted in the absence of any commercial or financial relationships that could be construed as a potential conflict of interest.
